# Lateral hypothalamus involvement in control of stress response by bed nucleus of the stria terminalis endocannabinoid neurotransmission in male rats

**DOI:** 10.1038/s41598-021-95401-z

**Published:** 2021-08-09

**Authors:** Lucas Gomes-de-Souza, Willian Costa-Ferreira, Michelle M. Mendonça, Carlos H. Xavier, Carlos C. Crestani

**Affiliations:** 1 Laboratory of Pharmacology, School of Pharmaceutical Sciences, São Paulo State University (UNESP), Araraquara, São Paulo Brazil; 2 Joint UFSCar-UNESP Graduate Program in Physiological Sciences, São Carlos, São Paulo Brazil; 3 Institute of Biological Sciences, Federal University of Goiás, Goiania, Goiás Brazil; 4 Laboratory of Pharmacology, Department of Department of Drugs and Pharmaceutics, School of Pharmaceutical Sciences, São Paulo State University - UNESP, Rodovia Araraquara-Jau Km 01 (Campus Universitário), Campus Ville, Araraquara, SP 14800-903 Brazil

**Keywords:** Neuroscience, Physiology

## Abstract

The endocannabinoid neurotransmission acting via local CB_1_ receptor in the bed nucleus of the stria terminalis (BNST) has been implicated in behavioral and physiological responses to emotional stress. However, the neural network related to this control is poorly understood. In this sense, the lateral hypothalamus (LH) is involved in stress responses, and BNST GABAergic neurons densely innervate this hypothalamic nucleus. However, a role of BNST projections to the LH in physiological responses to stress is unknown. Therefore, using male rats, we investigated the role of LH GABAergic neurotransmission in the regulation of cardiovascular responses to stress by CB_1_ receptors within the BNST. We observed that microinjection of the selective CB_1_ receptor antagonist AM251 into the BNST decreased the number of Fos-immunoreactive cells within the LH of rats submitted to acute restraint stress. Treatment of the BNST with AM251 also enhanced restraint-evoked tachycardia. Nevertheless, arterial pressure increase and sympathetically-mediated cutaneous vasoconstriction to restraint was not affected by CB_1_ receptor antagonism within the BNST. The effect of AM251 in the BNST on restraint-evoked tachycardia was abolished in animals pretreated with the selective GABA_A_ receptor antagonist SR95531 in the LH. These results indicate that regulation of cardiovascular responses to stress by CB_1_ receptors in the BNST is mediated by GABAergic neurotransmission in the LH. Present data also provide evidence of the BNST endocannabinoid neurotransmission as a mechanism involved in LH neuronal activation during stressful events.

## Introduction

The bed nucleus of the stria terminalis (BNST) has been implicated in physiological and behavioral responses to stress^1–3^. Regarding the cardiovascular responses, previous studies demonstrated that BNST modulates the blood pressure and heart rate (HR) increases caused by both unconditioned and conditioned stressfull stimuli, as well as by non-aversive environmental challenges (e.g., exercise)^4–6^.

Several neurochemical mechanisms have been implicated in the BNST control of stress responses^1,7^, including the endocannabinoid system^8^. Indeed, the presence of endocannabinoid receptors and enzymes involved in endocannabinoid synthesis and degradation were identified within the BNST^9–15^. Activation of BNST endocannabinoid neurotransmission during aversive threats was first evidenced by demonstration that systemic administration of a selective CB_1_ receptor antagonist enhanced BNST neuronal activation evoked by stress^16,17^. Accordingly, recent studies identified a role of BNST CB_1_ receptors in anxiogenic responses to stress^11,18^. We also reported an inhibitory influence of CB_1_ receptors present in the BNST in tachycardia observed during acute restraint stress^13^. Taken together, these results indicated the BNST endocannabinoid neurotransmission as part of the neural pathway regulating stress responses. However, the neural circuit related to this control is unknown.

The BNST is proposed as an important site connecting corticolimbic structures with effector nuclei of physiological and behavioral responses in the hypothalamus and brainstem^1–3,7^. In this sense, the BNST sends dense projections to the lateral hypothalamus (LH)^19,20^. Such as BSNT, the LH also plays a role in physiological and behavioral responses to emotional stress^2,21–24^. Regarding the stress-evoked cardiovascular responses, previous studies documented a role of this diencephalic region controlling cardiovascular responses evoked by both conditioned and unconditioned aversive stimuli^25–27^. The LH has an inhibitory influence in cardiovascular responses to unconditioned stress^27^, which is mediated by a balance of local excitatory and inhibitory inputs. Indeed, local LH treatment with a selective NMDA glutamatergic receptor antagonist enhanced the HR response to restraint stress^27^, whereas opposite effect was observed following LH treatment with a selective GABA_A_ receptor antagonist^28^.

The majority of neurons within the BNST present a GABAergic phenotype^29–32^. Accordingly, some studies provided evidence of GABAergic inputs within the LH arising from the BNST^33,34^. These morphofunctional evidence, taken together with evidence stated above of an inhibitory role of LH in tachycardia to restraint^27^, supported the idea that the LH might be part of the neural pathway related to the inhibitory control of restraint-evoked tachycardia by BNST endocannabinoid neurotransmission. In this sense, considering recent evidence that regulation of restraint-evoked tachycardia by BNST CB_1_ receptor is mediated by inhibition of local glutamatergic neurotransmission^35^, we investigated the hypothesis that the antagonism of CB_1_ receptor within the BNST decreases local neuronal activation within the LH as resulted of increased activation of BNST GABAergic neurons projecting to the HL, which in turn increases HR response to stress.

## Results

### Effect of CB_1_ receptor blockade within the BNST on number of Fos-positive neurons in the LH of stressed animals

**Figure 1:**
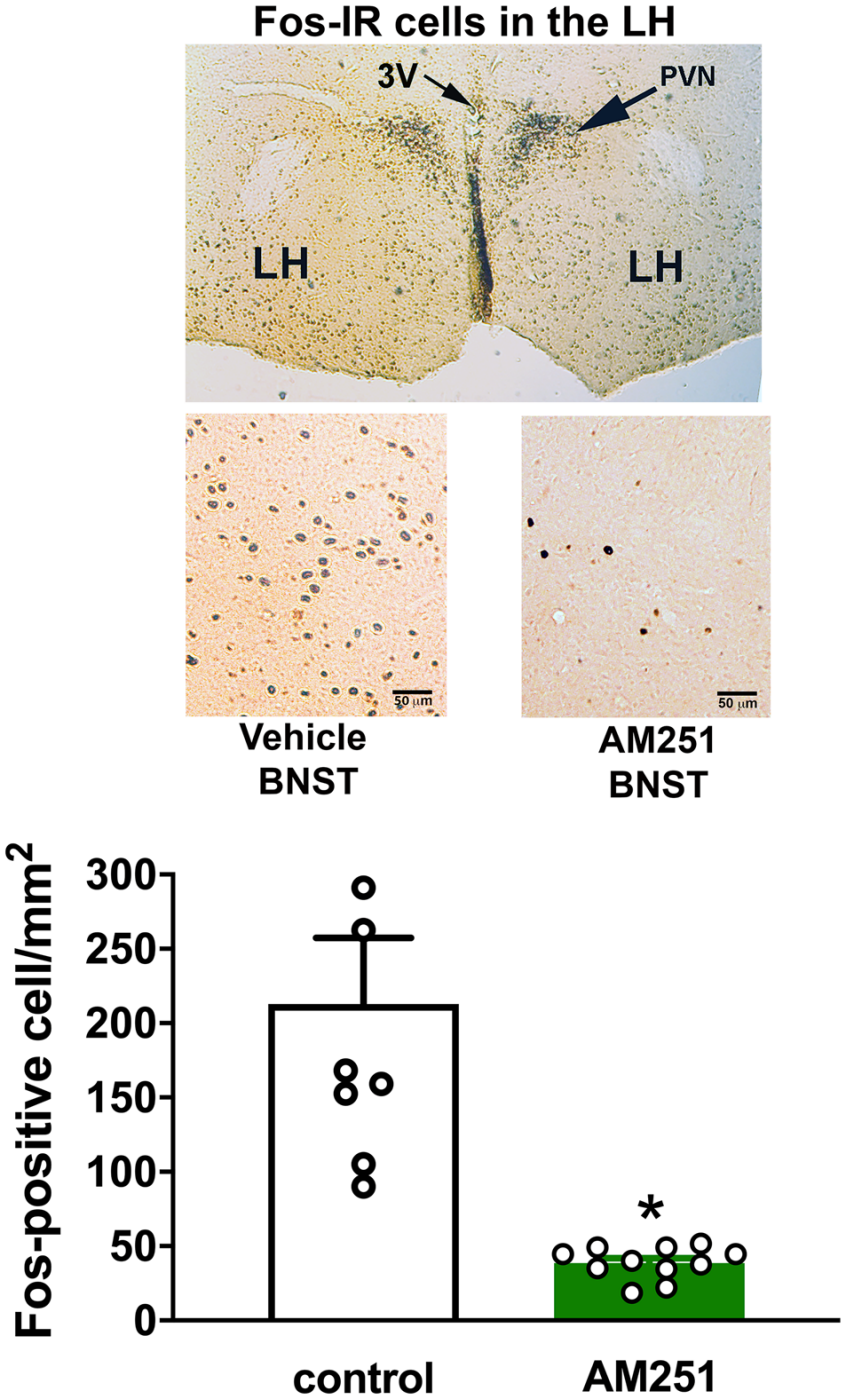
Effect of bed nucleus of the stria terminalis (BNST) treatment with the CB_1_ receptor antagonist AM251 in the number of Fos-immunoreactive (IR) cells in the LH following exposure to a 60-min session of restraint stress. (Top) Representative coronal sections showing Fos-IR cells in the LH following restraint stress exposure in animals that received bilateral microinjection of vehicle or AM251 into the BNST (bottom), as well as a representative section indicating the LH location (top). *3V* third ventricle, *LH* lateral hypothalamus, *PVN* paraventricular nucleus of the hypothalamus. (Bottom) Number of Fos-IR cells in the LH following exposure to acute restraint stress in animals treated with vehicle [solution of saline containing 30% of DMSO (DMSO), 100 nL, n = 8] (white bar) or the selective CB_1_ receptor antagonist AM251 (100 pmol/100 nL, n = 11) (green bar) into the BNST. The bars represent the mean ± SEM. * P < 0.05, Student's *t* test.

Bilateral microinjection of the selective CB_1_ receptor antagonist AM251 (100 pmol/100 nL/side, n = 11) into the BNST decreased the number of Fos-positive cells in the LH following exposure to restraint stress (t = 4.59; df = 17, P = 0.0003), when compared to vehicle-treated animals (100 nL/side, n = 8) (Fig. 1). Figure 1 also presents representative coronal sections of the LH region showing Fos-positive cells of animals subjected to restraint stress that received vehicle or AM251 into the BNST, as well as a representative section indicating the LH location.

### Effect of GABA_A_ receptor antagonism in the LH in changes on arterial pressure and HR reactivity to acute restraint stress evoked by CB_1_ receptor blockade in the BNST

Analysis of basal parameters (i.e., pre-stress values) indicated that bilateral microinjections of the GABA_A_ receptor antagonist SR95531 (1 pmol/100 nL/side) into the LH and/or the selective CB_1_ receptor antagonist AM251 (100 pmol/100 nL/side) into the BNST affected mean arterial pressure (MAP) (F_(3,24)_ = 3.3, P = 0.0376), but without changing HR (F_(3,24)_ = 0.5, P = 0.7259) (Table 1). Nevertheless, post-hoc analysis of MAP basal values did not reveal specific differences between the experimental groups (P > 0.05) (Table 1).

**Table 1:** Basal parameters of mean arterial pressure (MAP), heart rate (HR) and tail skin temperature (T) after pharmacological treatment of the BNST with the selective CB_1_ receptor antagonist AM251 (or vehicle) and/or the LH with the selective GABA_A_ receptor antagonist SR95531 (or vehicle).

Groups	n	MAP (mmHg)	HR (bpm)	T (ºC)
SAL LH + DMSO BNST	7	109 ± 2	392 ± 8	28.7 ± 0.2
SAL LH + AM251 BNST	7	111 ± 3	389 ± 11	29.1 ± 0.1
SR LH + DMSO BNST	7	104 ± 1	398 ± 8	29.6 ± 0.2
SR LH + AM251 BNST	7	104 ± 2	406 ± 16	28.9 ± 0.5

Values are mean ± SEM, one-way ANOVA.

*DMSO* saline containing 30% of DMSO, *SAL* saline, *SR* SR95531.

**Figure 2:**
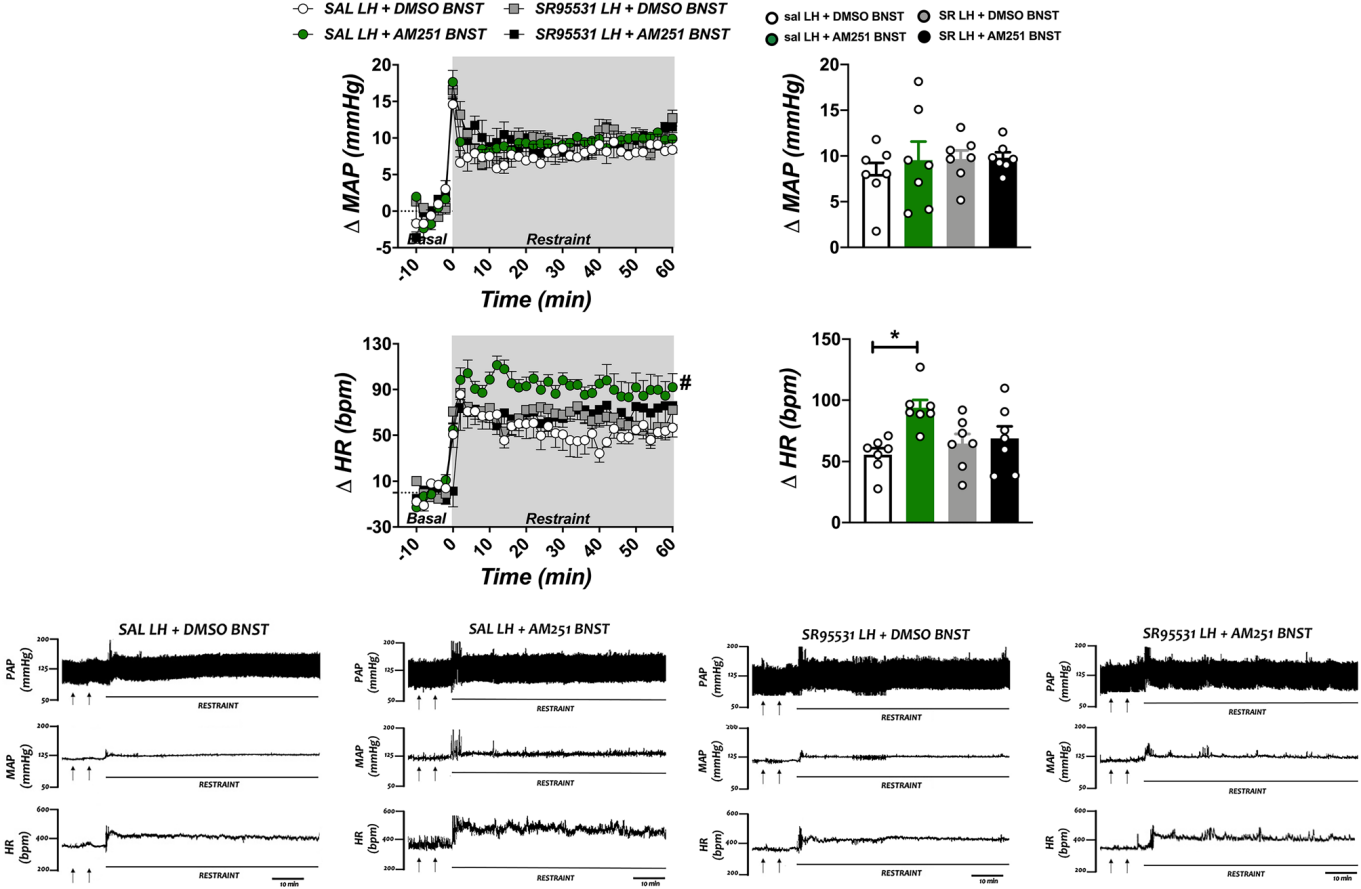
Effect of lateral hypothalamus (LH) treatment with the selective GABA_A_ receptor antagonist SR95531 and/or microinjection of the CB_1_ receptor antagonist AM251 into the bed nucleus of the stria terminalis (BNST) in arterial pressure and heart rate (HR) changes evoked by an acute session of restraint stress. (Top, left) Time-course curves of changes on mean arterial pressure (Δ MAP) and HR (Δ HR) evoked by acute restraint stress in animals treated bilaterally into the LH with saline (SAL, 100 nL) or the selective GABA_A_ receptor antagonist SR95531 (1 pmol/100 nL), followed by a second microinjection into the BNST of vehicle [solution of saline containing 30% of DMSO (DMSO), 100 nL] or the selective CB_1_ receptor antagonist AM251 (AM251, 100 pmol/100 nL). Circles represent the mean ± SEM. ^#^P < 0.05 over the entire restraint period compared to SAL LH + DMSO BNST group. Two-way ANOVA accompanied by Bonferroni post-hoc test (n = 7/group). (Top, right) Mean Δ MAP and Δ HR during the entire restraint stress period in animals treated bilaterally into the LH with SAL (100 nL) or SR95531 (1 pmol/100 nL), followed by a second microinjection into the BNST of DMSO (100 nL) or AM251 (100 pmol/100 nL). Columns represent the mean and bars the SEM. *P < 0.05 in relation to SAL LH + DMSO BNST group. One-way ANOVA accompanied by Bonferroni post-hoc test (n = 7/group). (Bottom) Pulsatile arterial pressure (PAP), MAP and HR recordings before and during restraint stress of representative rats illustrating the effect of local LH treatment with saline (SAL) or the selective GABA_A_ receptor antagonist SR95531, followed by a second microinjection into the BNST of vehicle (DMSO) or the selective CB_1_ receptor antagonist AM251 (AM251). The arrows indicate the microinjection into the LH and BNST, respectively. Note the increase in restraint-evoked tachycardia in SAL LH + AM251 BNST group, which was inhibited when the LH was pretreated with the GABA_A_ receptor antagonist (SR95531 LH + AM251 BNST group).

Analysis of the time-course curves indicated that acute restraint stress caused a sustained increase on both MAP (time factor: F_(35,840)_ = 54, P < 0.0001) and HR (time factor: F_(35,840)_ = 56, P < 0.0001), (Fig. 2). Two-way ANOVA also indicated effect of BNST and/or LH pharmacological treatments on restraint-evoked HR increase (F_(3,24)_ = 4.0, P = 0.0198), but without affecting MAP (F_(3,24)_ = 0.4, P = 0.7551) (Fig. 2). A treatment × time interaction for HR (F_(105,840)_ = 2.4, P < 0.0001) and MAP (F_(105,840)_ = 1.5, P = 0.0042) was also evidenced. Post-hoc analysis revealed that AM251 into the BNST (saline LH + AM251 BNST group) increased restraint-evoked tachycardiac response (P = 0.0077) (Fig. 2). The effect of AM251 within the BNST on HR increase to restraint stress was inhibited by LH pretreatment with the GABA_A_ receptor antagonist (SR95531 LH + AM251 BNST group) (P = 0.5898) (Fig. 2). Post-hoc analyisis did not reveal specific differences between the experimental groups on MAP response (P > 0.05) (Fig. 2).

Analysis of the mean change during the entire restraint period indicated effect of pharmacological treatments on HR increase (F_(3,24)_ = 4.7, P = 0.0103), but without affecting MAP response (F_(3,24)_ = 0.4, P = 0.7553) (Fig. 2). Post-hoc analysis revealed that AM251 into the BNST (sal LH + AM251 BNST group) increased the tachycardia to restraint stress (P = 0.0042), and such potentiation effect was absent in animals pretreated with the GABA_A_ receptor antagonist into the LH (SR95531 LH + AM251 BNST group) (P = 0.4760) (Fig. 2).

Figure 2 presents representative experimental recordings showing the effect of restraint stress in MAP and HR in animals that received vehicle or the selective GABA_A_ receptor antagonist into the LH, followed by microinjection of vehicle or the CB_1_ receptor antagonist into the BNST.

### Effect of GABA_A_ receptor antagonism in the LH in changes on tail skin temperature reactivity to acute restraint stress evoked by CB_1_ receptor blockade in the BNST

**Figure 3:**
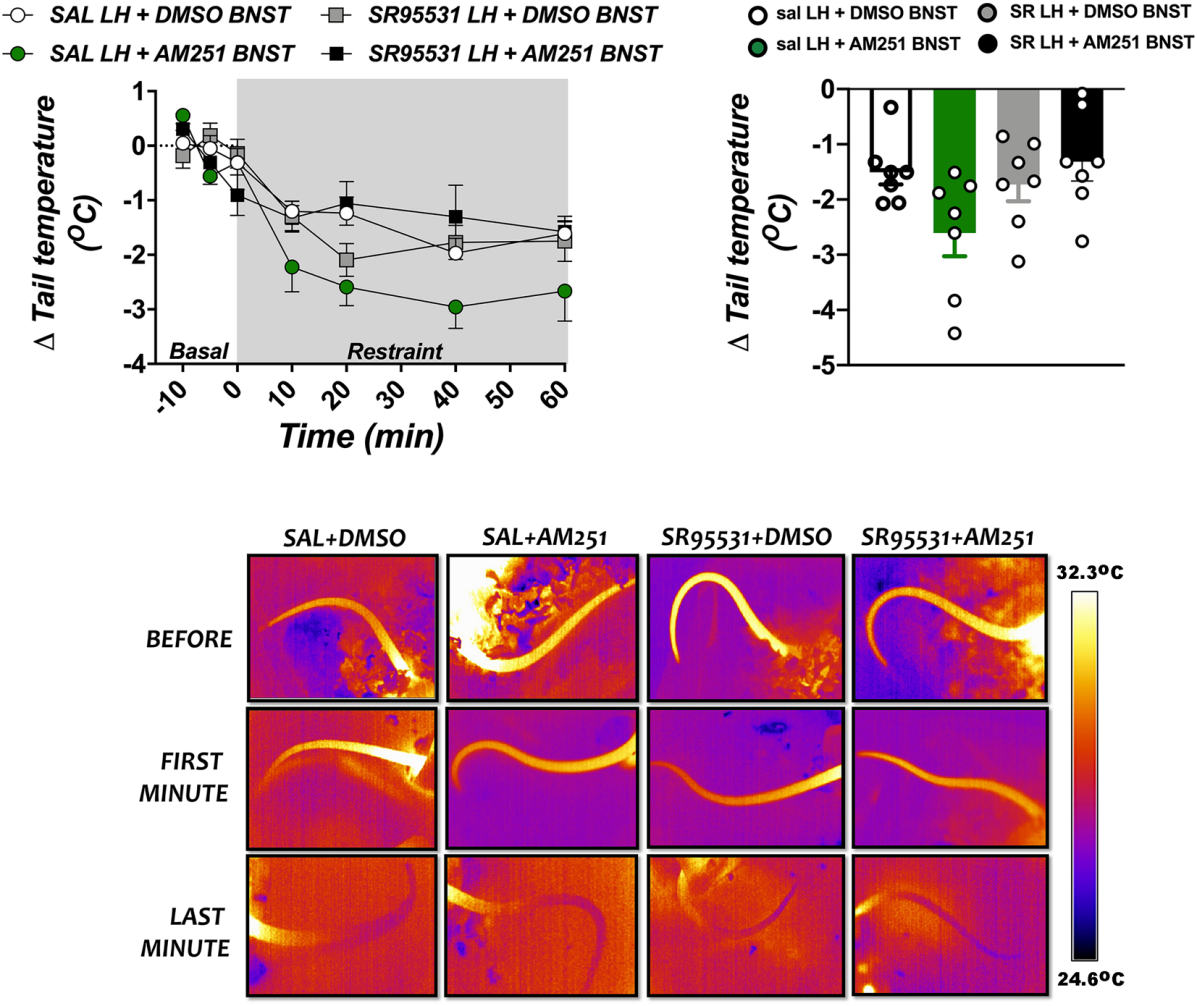
Effect of lateral hypothalamus (LH) treatment with the selective GABA_A_ receptor antagonist SR95531 and/or microinjection of the CB_1_ receptor antagonist AM251 into the bed nucleus of the stria terminalis (BNST) in drop in tail skin temperature evoked by an acute session of restraint stress. (Top, left) Time-course curves of changes in tail skin temperature (Δ tail temperature) evoked by acute restraint stress in animals treated bilaterally into the LH with saline (SAL, 100 nL) or the selective GABA_A_ receptor antagonist SR95531 (1 pmol/100 nL), followed by a second microinjection into the BNST of vehicle [solution of saline containing 30% of DMSO (DMSO), 100 nL] or the selective CB_1_ receptor antagonist AM251 (AM251, 100 pmol/100 nL). Circles represent the mean ± SEM. Two-way ANOVA (n = 7/group). (Top, right) Mean Δ tail temperature during the entire restraint stress period in animals treated bilaterally into the LH with SAL (100 nL) or SR95531 (1 pmol/100 nL), followed by a second microinjection into the BNST of DMSO (100 nL) or AM251 (100 pmol/100 nL). Columns represent the mean and bars the SEM. One-way ANOVA (n = 7/group). (Bottom) Images of representative rats showing the tail skin temperature before and at first and last minute of restraint stress in animals treated with saline (SAL) or the selective GABAA receptor antagonist SR95531 into the LH, followed by a second microinjection of vehicle (DMSO) or the selective CB_1_ receptor antagonist AM251 (AM251) into the BNST. Note the absence of effect of the pharmacological treatments.

Bilateral microinjections of the GABA_A_ receptor antagonist SR95531 (1 pmol/100 nL/side) into the LH and/or the selective CB_1_ receptor antagonist AM251 (100 pmol/100 nL/side) did not affect the basal values (i.e., pre-stress level) of tail skin temperature (F_(3,24)_ = 1.9, P = 0.1656) (Table 1). However, analysis of the time-course curves indicated that acute restraint stress decreased the skin temperature (time factor: F_(6,144)_ = 53, P < 0.0001) (Fig. 3). Two-way ANOVA did not indicate effect of BNST and/or LH pharmacological treatments on restraint-evoked decrease in tail skin temperature (F_(3,24)_ = 1.8, P = 0.1847) (Fig. 3), but a treatment x time interaction was evidenced (F_(18,144)_ = 3.1, P = 0.0059). Nevertheless, post-hoc analysis did not reveal difference between the experimental groups in restraint-evoked drop in tail skin temperature (P > 0.05) (Fig. 3). Analysis of the mean change during the entire restraint period also did not indicate effect of pharmacological treatments on tail skin temperature response (F_(3,24)_ = 2.9, P = 0.0515) (Fig. 3). Figure 3 presents representative images showing the tail skin temperature before and during restraint stress in animals that received vehicle or the selective GABA_A_ receptor antagonist into the LH, followed by microinjection of vehicle or the CB_1_ receptor antagonist into the BNST.

## Discussion

The present results indicate for the first the LH as part of the neural pathway regulating physiological responses during stressful events by the BNST. In fact, we observed that BNST treatment with the selective CB_1_ receptor antagonist AM251 into the BNST facilitated the tachycardia evoked by restraint stress, but without affecting the pressor and sympathetically-mediated cutaneous vasoconstriction. The facilitatory influence of CB_1_ receptor antagonism within the BNST on restraint-evoked tachycardia was completely inhibited in animals pretreated in the LH with the selective GABA_A_ receptor antagonist SR95531. Besides, we identified that bilateral microinjection of AM251 into the BNST decreased the number of Fos-immunoreactive cells in the LH of animals subjected to restraint stress.

We reported previously that microinjection of the selective CB_1_ receptor antagonist AM251 into the BNST dose-dependently enhanced the tachycardia (without affecting blood pressure and tail skin temperature responses) observed during acute restraint stress^13^. Conversely, increase in either anandamide or 2-arachidonoylglycerol levels in the BNST decreased HR response to restraint stress, and the effect of both endocannabinoids were inhibited in animals pretreated in the BNST with AM251^13^. These previous results support the present findings indicating an inhibitory role of CB_1_ receptors in restraint-evoked tachycardia.

CB_1_ receptors are expressed predominantly in presynaptic terminals^36–38^. Accordingly, CB_1_ receptor was identified in both excitatory and inhibitory terminals onto BNST neurons, and its activation inhibited local glutamatergic and GABAergic inputs^14,15^. However, CB_1_ receptor activation present in glutamatergic terminals seem to be prominent during aversive threats within the BNST. For instance, previous findings identified that CB_1_ receptor blockade enhanced stress-evoked c-fos mRNA in the BNST^16,17^. Besides, we reported recently that the facilitated tachycardia to restraint stress following BNST treatment with AM251 was inhibited by local NMDA glutamate receptor antagonism within the BNST^35^. The idea that control of cardiovascular responses to restraint by BNST CB_1_ receptor is mediated by interaction with local glutamatergic neurotransmission is further supported by evidence that BNST NMDA glutamate receptor plays a facilitatory influence in restraint-evoked HR response without affecting pressor and tail skin temperature changes^39,40^.

**Figure 4:**
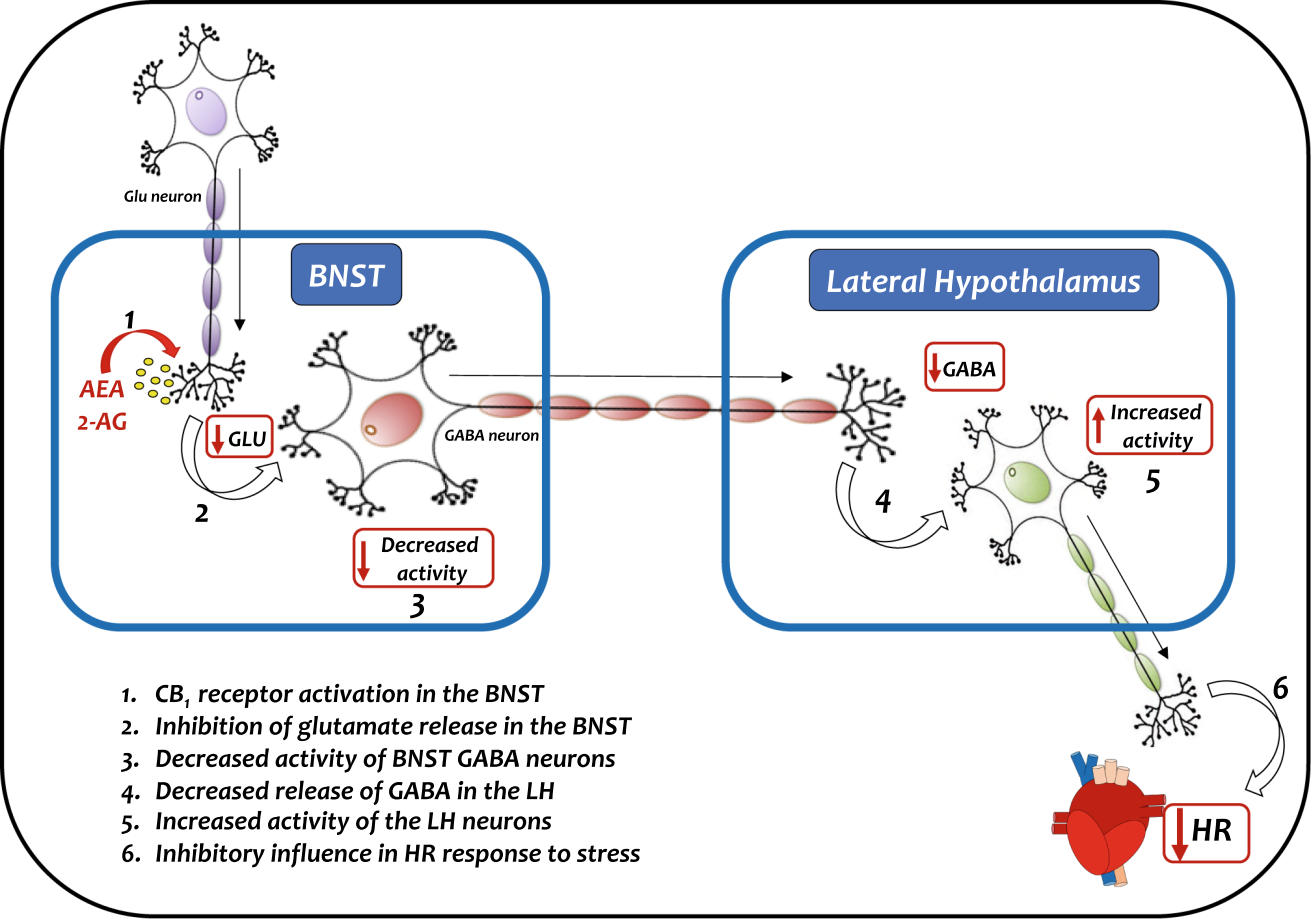
Schematic representation illustrating the proposed mechanism of interaction between BNST endocannabinoid neurotransmission acting via local CB_1_ receptor and GABAergic neurotransmission within the LH in the control of tachycardiac response during aversive threats. Activation of CB_1_ receptor in the BNST present in glutamatergic terminals (1) inhibits the local release of glutamate (2), which in turn decreases the activity of BNST GABA neurons (3) and, consequently, the release of GABA in the HL (4). The decreased GABA release increase activity of local HL neurons (5), which cause an inhibitory influence in HR increase during stresstul events (6) (*please, see the text for details*). *2-AG* 2-arachidonoylglycerol, *AEA* anandamide, *BNST* bed nucleus of the stria terminalis. *Glu* glutamate, *HR* heart rate.

Current data provide evidence regarding the neural circuit related to the control of tachycardia to restraint stress by BNST CB_1_ receptor by indicating a prominent role of projections to the LH. As stated in the Introduction, previous studies provided evidence of GABAergic inputs within the LH arising from the BNST^33,34^. These neuroanatomical evidence, taken together with results mentioned above that CB_1_ receptor activation during aversive threats acts mainly inhibiting glutamatergic terminals during aversive threats^16,17,35^ indicate that the decrease in number of Fos-immunoreactive cells in the LH observed in the present study in animals treated with the CB_1_ receptor antagonist in the BNST might be the resulted of an increased local glutamatergic neurotransmission, which in turn increase activation of BNST GABAergic neurons projecting to the LH. The hypothesis that GABAergic connection with the LH mediates the inhibitory control of BNST CB_1_ receptor was further supported by demonstration that LH pretreatment with the GABA_A_ receptor antagonist SR95531 completely inhibited the facilitation of HR increase to restraint stress caused by BNST treatment with the CB_1_ receptor antagonist AM251. Therefore, the amplitude of the tachycardia evoked by acute stress exposure rely on CB_1_ receptor within the BNST governing reduction in GABAergic influence exerted by BNST upon LH neurons, which in turn increase activity of LH neurons inhibiting heartbeat during stressful event. This idea is in line with recent report that GABA_A_ receptor antagonism within the LH decreased restraint-evoked tachycardia^28^. Besides, a previous study documented that inhibition of BNST GABAergic terminals within the LH caused increase in local postsynaptic neuronal activity^34^. Figure 4 shows schematic representation illustrating the proposed mechanism involving BNST endocannabinoid neurotransmission and LH GABAergic mechanism for the control of HR response during stressful events.

GABAergic projections from the BNST to the LH have been previously implicated in behavioral responses, including feeding and anxiety-like behavior^34,41^. However, this pathway seem not to be related to the control of cocaine conditioned place preference by LH orexin neurons^42^. Besides, the decrease in anxiety-like behaviors evoked by stimulation of BNST-LH projection was not followed by changes in respiratory rate, which indicated that this neural network was not involved in physiological changes related to this behavioral response^41^. Therefore, to the best of our knowledge, our findings are the first to indicate an involvement of the BNST-LH pathway in physiological responses during aversive threats^7,43^. Besides, in addition to provide evidence of the brain network involved in the control of cardiovascular responses by BNST endocannabinoid neurotransmission, results reported here also indicate a mechanism involved in neuronal activation within the LH during aversive threats.

GABAergic projections from the BSNT targets glutamatergic neurons within the LH^34^. Previous studies identified inputs from the LH in parasympathetic brainstem nuclei^44,45^. Indeed, Deolindo et al.^27^ reported that cardiac parasympathetic activity governs the inhibitory control of restraint-evoked HR increase by the LH. Control of tachycardia to restraint by the BNST was also documented to be mediated by facilitation of parasympathetic nervous system^5^. Based on these pieces of evidence, it is possible that the inhibitory control of restraint-evoked HR increase by BNST CB_1_ receptor occouring via inhibition of GABAergic inputs within the LH is mediated by an increase in activity of LH glutamatergic neurons projecting to parasympathetic centers in the medulla. However, BNST projections also target neurons expressing orexin and melanin concentrating hormone (MCH) within the LH^7,20^. Although evidence that BNST GABAergic neurons inhibits MCH cells within the LH^20^, these neurons are inhibited by aversive stimuli^20^, which preclude the idea of a role of MCH cells in control of stress responses by BNST CB_1_ receptors. Orexin neurons also seem not to be part of the pathway proposed in the present study once this neurochemical mechanism in the brain plays a facilitatory influence in stress-evoked cardiovascular changes^46–48^, so that increased activity of LH orexin neurons would increase rather than decrease restraint-evoked HR increase. The absence of an involvement of orexin neurons is further supported by evidence that orexinergic mechanisms are not involved in cardiovascular changes caused by restraint^47^.

The idea that control of tachycardia to stress by BNST CB_1_ receptors is mediated by direct GABAergic projections to the LH is supported by evidence of the BNST as a prominent source of GABAergic inputs in the LH^20,33^. Besides, previous studies have indicated that optogenetic activation of BNST GABAergic terminals within the LH affected feeding and anxiety-like behaviors^34,41^. However, we cannot exclude the possibility that involvement of LH GABAergic neurotransmission in BNST CB_1_ receptor control of tachycardia to restraint stress is mediated by recruitment of intermediate brain regions. In fact, previous studies identified LH-projecting GABAergic neurons in brain regions that receive BNST inputs^49–52^ and are involved in control of stress-evoked cardiovascular responses^53–55^, such as amygdala and lateral preoptic area. Therefore, further studies are necessary to directly assess if control of cardiovascular responses to stress by BNST CB_1_ receptors are mediated by direct or indirect connections with the LH.

In summary, the results reported in the present study provide evidence of the LH as part of the neural network regulating the cardiovascular responses to aversive threats by BNST endocannabinoid neurotransmission. In fact, our data indicate that the inhibitory control related to CB_1_ receptors activation within the BNST in tachycardia to stress is mediated by LH GABAergic neurotransmission acting via local GABA_A_ receptors. Findings reported here also provide evidence that BNST endocannabinoid neurotransmission is potentially involved in activation of LH neurons during aversive threats.

## Methods

### Animals

Forty-seven male Wistar rats weighting 240–260 g (60-days-old) were used. Animals were obtained from the animal breeding facility of the São Paulo State University (UNESP) (Botucatu, SP, Brazil), and were housed according to conditions stablished in our laboratory^13,28,56,57^. Briefly, the rats were housed in plastic cages in a temperature-controlled room at 24 °C in the Animal Facility of the Laboratory of Pharmacology (School of Pharmaceutical Sciences/UNESP). They were kept under a 12:12 h light–dark cycle (lights on between 7:00 am and 7:00 pm) with free access to water and standard laboratory food. Housing conditions and experimental procedures were approved by the Ethical Committee for Use of Animals of the School of Pharmaceutical Sciences/UNESP (approval # 61/2015), which complies with Brazilian and international guidelines for animal use and welfare. The study was carried out in compliance with the ARRIVE guidelines.

### Implant of brain cannulas

Five days before the trial, rats were anesthetized with tribromoethanol (250 mg/kg, i.p.). After scalp anesthesia with 2% lidocaine, the skull was exposed and stainless-steel guide cannulas (26 G, 12 mm-long) directed to the LH and/or BNST were bilaterally implanted at a position 1 mm above the site of injection, using a stereotaxic apparatus (Stoelting, Wood Dale, IL, USA). Stereotaxic coordinates for cannula implantation into the BNST were: antero-posterior =  + 7.8 mm from interaural line; lateral = 4 mm from the medial suture; dorso-ventral =  − 5.8 mm from the skull, with a lateral inclination of 23°^58^. For the LH, the stereotaxic coordinates were: antero-posterior =  + 6.2 mm from interaural line; lateral = 1.8 mm from the medial suture; dorso-ventral =  − 7.6 mm from the skull; without lateral angulation^58^. Cannulas were fixed to the skull with dental cement and one metal screw. After the surgery, all animals received a poly-antibiotic solution containing streptomycins and penicillins (560 mg/ml/kg, i.m.) to prevent infection and the non-steroidal anti-inflammatory flunixin meglumine (0.5 mg/ml/kg, s.c.) for post-operation analgesia.

### Cannulation of femoral artery

One day before the trial, rats were anesthetized with tribromoethanol (250 mg/kg, i.p.), and a catheter (Clay Adams, Parsippany, NJ, USA) filled with a solution of heparin (50UI/ml, Hepamax-S, Blausiegel, Cotia, SP, Brazil) diluted in saline (0.9% NaCl) was inserted into the abdominal aorta through the femoral artery for cardiovascular recording, according to procedures previously described by our group^13,57,59,60^. After the surgery, the non-steroidal anti-inflammatory flunixin meglumine (0.5 mg/ml/kg, s.c.) was administered for post-operation analgesia. The animals were kept in individual cages during the postoperative period and cardiovascular recording.

### Restraint stress

The acute restraint stress consisted of introducing the animals into plastic cylindrical tubes (diameter = 6.5 cm, length = 15 cm), which were ventilated by ½ inch holes that comprised approximately 20% of the tube, as previously described by us^5,13^. The animals were maintained for a period of 60 min into the restraint tube^5,13,27^. Each animal was submitted to only one session of stress in order to avoid habituation^61–63^.

### Blood pressure and heart rate recording

The catheter implanted into the femoral artery was connected to a pressure transducer (DPT100, Utah Medical Products Inc., Midvale, UT, USA), and pulsatile arterial pressure (PAP) was recorded using an amplifier (Bridge Amp, ML224, ADInstruments, Australia) and an acquisition board (PowerLab 4/30, ML866/P, ADInstruments, NSW, Australia) connected to a personal computer, as previously described by us^13,57,64,65^. Mean arterial pressure (MAP) and HR values were derived from the PAP recording.

### Tail cutaneous temperature measurement

Vasomotor sympathetic activity activation during aversive threats decreases cutaneous blood flow^66^ that in turn reduces cutaneous temperature^67^. Therefore, the drop in tail cutaneous temperature was evaluated as an indirect measurement of vasomotor sympathetic response in cutaneous beds during restraint stress^13,35,67^.

The tail cutaneous temperature was recorded using a thermal camera (IRI4010, Infra Red Integrated Systems Ltd., Northampton, UK). The analysis was performed using a software for thermographic analysis, and temperature was represented by color intensity variations^67,68^. For image analysis, the temperature was measured on five points along the animal’s tail, and the mean value was calculated for each recording^13,35,39,69^.

### Drug microinjection

The needles (33G, Small Parts, Miami Lakes, FL, USA) used for microinjection into the BNST and/or LH were 1 mm longer than the guide cannulas and were connected to a 2 μL syringe (7002-KH, Hamilton Co., Reno, NV, USA) via a PE-10 tubing (Clay Adams, Parsippany, NJ, USA). Intra-cerebral microinjections were performed within a 5 s period, and the needle was left in the guide cannula for 1 min after the microinjection before being removed. Microinjection was performed without restraining the animals, and drugs were administrated into the LH and BNST in a final volume of 100 nL per side^5,13,27,28,67^.

### Drugs and solutions

SR95531 (selective GABA_A_ receptor antagonist) (TOCRIS, West-woods Business Park, Ellisville, MO, USA; cat. #1262), 2,2,2-tribromoethanol (Sigma-Aldrich, St Louis, Missouri, USA; cat. #T48402) and urethane (Sigma-Aldrich; cat. #U2500) were dissolved in saline (NaCl 0.9%). AM251 (N-(piperidin-1-yl)-5-(4-iodophenyl)-1-(2,4-dichlo-rophenyl)-4-methyl-1H-pyrazole-3 carboxamide) (selective CB_1_ receptor antagonist) (TOCRIS, cat. #1117) was dissolved in a solution of saline containing 30% of DMSO (DMSO). Flunixin meglumine (Banamine, Schering Plough, Cotia, SP, Brazil) and the polyantibiotic preparation of streptomycins and penicillins (Pentabiotico, Fort Dodge, Campinas, SP, Brazil) were used as provided.

### Immunohistochemistry

Thirty minutes after the end of the stress session, the animals were anesthetized with urethane (1.2 g/kg,i.p.) and perfused with saline phosphate (PBS) (1X pH 7.4) accompanied by 4% paraformaldehyde in solution with phosphate buffer (pH 7.4). Then, the brain was removed and post-fixed in paraformaldehyde for 2 h and transferred to 30% sucrose solution in PBS at 4 °C. Two days later, the brains were frozen in dry ice powder for 1 h, and then stored in freezer at − 80 °C until processing.

Before the imunnohistochemistry procedures, the brains were sectioned in a cryostat (− 20 °C) (CM1900, Leica, Germany) with a thickness of 35 μm according to coodinates of Paxinos and Watson^58^. The slices containing the LH region were washed 3 times (10 min each wash) in PBS and incubated in blocking solution (3% goat serum and 0.25% Triton X-100) dissolved in PBS for one hour at room temperature. After the blockage, the slices were incubated with anti- Fos primary antibody (1:2000 dilution; Cell Signaling Technology, Danvers, MA, USA; produced in rabbit) for 24 h at 4 °C. After the incubation, the slices were washed with PBS 3 times (10 min each wash) and incubated with biotinylated anti-rabbit secondary antibody (dilution 1: 600; Vector Laboratories, Burlingame, CA, USA) in PBS-Tx (0.25% Triton X-100) and 3% goat serum for 2 h at room temperature. The slices were then washed with PBS 3 times (10 min each wash) and incubated for 1 h in avidin–biotin-peroxidase solution (ABC Elite kit, PK-6100; Vector Laboratories, Burlingame, CA, USA), 0.5% Triton X-100 and PBS. The slices were then washed 3 times (10 min each wash) and incubated in 3,3′-diaminobenzidine (DAB) for seven min. Then, they were washed 4 times (5 min each wash), transferred to PBS solution and mounted on gelatinized slides. After drying, the slides were hydrated in distilled water and then gradient dehydrated by increasing ethanol titrations (30%, 60%, 90%, 95% and 100%) and xylol (LabSynth, São Paulo, Brazil). Finally, they were covered with Permount (Sigma-Aldrich, St. Louis, MA, USA) and coverslips.

Immunostaining of Fos was captured in a microscope coupled to a camera (Zeiss Axioskop 2). Two images were captured per slice (right and left hemispheres) and at least two slices were obtained per animal, and the counting was performed from a fixed area of the LH. The Fos-positive cells were counted using the ImageJ software (version 1.52q; website: https://imagej.nih.gov/ij/download.html). The results were expressed as mean number of Fos-positive cells/mm^2^. The LH was identified according to the atlas of rat`s brain of Paxinos and Watson^58^.

### Experimental design

Experimental procedures were as previously described by us^13,35,70^. Briefly, animals were brought to the experimental room in their own cages. Animals were allowed at least 60 min to adapt to the experimental room conditions, such as sound and illumination, before starting the experiments. The experimental room was temperature controlled (24 °C) and acoustically isolated from the other rooms.

#### Effect of CB_1_ receptor blockade within the BNST on number of Fos-positive neurons in the LH of stressed animals

This protocol aimed to test the hypothesis that antagonism of CB_1_ receptor within the BNST decreases the number of Fos-positive neurons in the LH during restraint stress. For this, animals were treated with either vehicle (saline containing 30% of DMSO, 100 nL/side, n = 8) or the selective CB_1_ receptor antagonist AM251 (100 pmol/100 nL/side, n = 11) into the BNST^13,35^. Ten min after the treatment, all rats were submitted to a 60 min session of restraint stress. Thirty minutes after the end of the stress session, the animals were anesthetized with urethane (1.2 g/kg,i.p.), perfused, and their brains were processed for immunohistochemistry protocol.

#### Effect GABA_A_ receptor antagonism in the LH in changes of cardiovascular and tail skin temperature reactivity to acute restraint stress evoked by CB_1_ receptor blockade in the BNST

The aim of this protocol was to evaluate the involvement GABAergic neurotransmission in the LH, acting via local GABA_A_ receptors, in changes of restraint-evoked MAP and HR increase and drop in tail skin temperature caused by CB_1_ receptor antagonism in the BNST. For this, independent sets of rats were pretreated into the LH with either the selective GABA_A_ receptor antagonist SR95531 (1 pmol/100 nL) or vehicle (saline, 100 nL/side)^28^. Five minutes later, the animals received either vehicle (saline containing 30% of DMSO, 100 nL/side) or AM251 (100 pmol/100 nL/side) into the BNST (n = 7/group, Table 1)^13,35^. Five minutes after the second pharmacological treatment, animals in all experimental groups underwent a 60 min session of restraint stress.

Blood pressure and HR recording started at least 30 min before the onset of the restraint, and was performed throughout the stress session. The tail skin temperature was measured 10, 5 and 0 min before the restraint for baseline values, and at 10, 20, 40 and 60 min during restraint^13,35^. Each animal received a single pharmacological treatment and was submitted to one session of restraint. In each protocol, animals were randomly distributed among the several experimental groups.

### Histological determination of the microinjection sites

At the end of each experiment, animals were anesthetized with urethane (1.2 g/kg, i.p.), and 1% Evan’s blue dye was microinjected into the brain at the same volume of drug injection (i.e.,100 nL/side) as a marker of microinjection site. Then, the brains were removed and post-fixed in 10% formalin solution for at least 48 h at 4 °C. Afterwards, serial 40 μm thick sections of the BNST region were cut using a cryostat (CM1900, Leica, Wetzlar, Germany) for identification of the microinjection sites according to Paxinos and Watson^58^.

**Figure 5:**
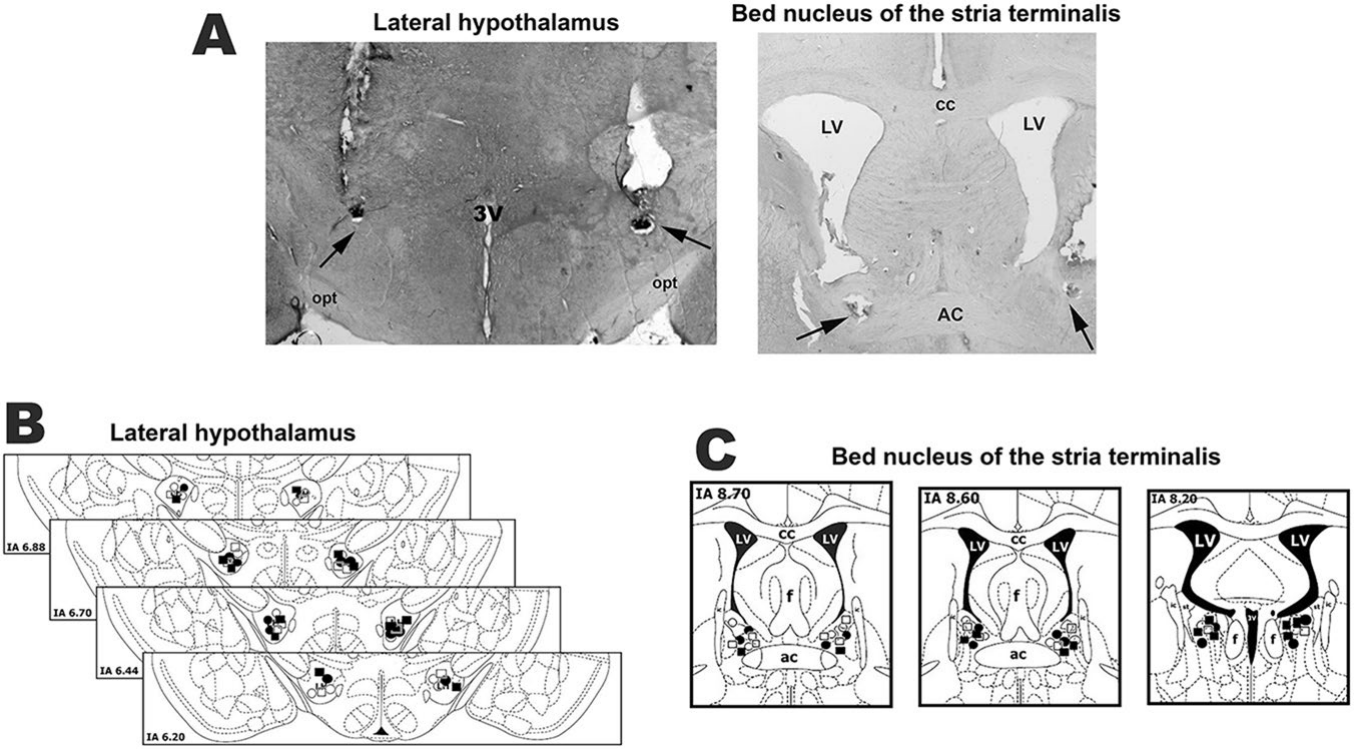
(**A**) Photomicrographs of coronal brain sections from representative rats showing bilateral sites of microinjection into the lateral hypothalamus (LH) (left) and bed nucleus of the stria terminalis (BNST) (right). (**B**, **C**) Diagrammatic representations based on the rat brain atlas of Paxinos and Watson^58^ indicating the microinjection sites into the lateral hypothalamus (**B**) and BNST (**C**) of all animals used for evaluation of the involvement of GABA_A_ receptor within the LH in cardiovascular changes evoked by microinjection of the selective CB_1_ receptor antagonist AM251 into the BNST. White circles: saline LH + DMSO BNST group; black circles: saline LH + AM251 BNST group; white squares: SR95531 LH + DMSO BNST group; black squares: SR95531 LH + AM251 BNST group. *3V* third ventricle, *ac* anterior commissure, *cc* corpus callosum, *f* fornix, *IA* interaural coordinate, *ic* internal capsule, *LV* lateral ventricle, *opt* optic chiasm, *st* stria terminalis.

Photomicrographs of coronal brain sections depicting bilateral microinjection sites in the LH and BNST of representative animals are presented in Fig. 5. Diagrammatic representations based on the brain atlas of Paxinos and Watson^58^ indicating the microinjection sites into the LH and BNST of all animals used in the present study are also presented in Fig. 5.

### Data analysis

Data were expressed as mean ± SEM. The number of Fos-positive cells in the LH were compared using the Student’s t-test. The basal values of MAP, HR and tail skin temperature were compared using one-way ANOVA followed by Bonferroni’s post-hoc test. Restraint-evoked cardiovascular changes were obtained by calculating the difference between the values recorded during the restraint stress and the baseline value obtained by the mean of points recorded across the 10 min before the restraint onset. The time-course curves of MAP, HR and tail skin temperature changes were analyzed using two-way ANOVA, with treatment as main factor and time as repeated measurement, followed the by the Bonferroni`s post-hoc test. The mean of the values during the entire restraint session was also calculated, and these values were compared using one-way ANOVA followed by Bonferroni’s post-hoc test. Results of statistical tests with P < 0.05 were considered significant.

## Data Availability

Data available on request from the authors.
